# Hard Chrome-Coated and Fullerene-Doped Metal Surfaces in Orthopedic Bearings

**DOI:** 10.3390/ma10121449

**Published:** 2017-12-20

**Authors:** Robert Sonntag, Katja Feige, Claudia Beatriz dos Santos, Jan Philippe Kretzer

**Affiliations:** 1 Laboratory of Biomechanics and Implant Research, Clinic for Orthopedics and Trauma Surgery, Heidelberg University Hospital, 69118 Heidelberg, Germany; philippe.kretzer@med.uni-heidelberg.de; 2 Fraunhofer Institute for Manufacturing Engineering and Automation IPA, Department Electroplating, 70569 Stuttgart, Germany; katja.feige@ipa.fraunhofer.de; 3 Institute of Industrial Manufacturing and Management IFF, University of Stuttgart, 70569 Stuttgart, Germany; cvs@iff.uni-stuttgart.de

**Keywords:** total hip arthroplasty, coating, chromium, electroplating, co-deposition, fullerene, C_60_, wear

## Abstract

Metal-on-metal bearings for total hip replacements have been introduced as an alternative to polyethylene in young and more active patients. These have, however, been shown to be prone to implant malpositioning and have been limited by some specific design features. In that context, coatings present an option to increase wear resistance by keeping the high fracture strength of the metal substrate. A custom-made electroplating setup was designed for the coating of CoCr substrates using (a) an industrial standard chromium electrolyte; (b) a custom-made hexavalent chromium (Cr^6+^) electrolyte with a reduced chromium trioxide (CrO_3_) content, both without solid additives and (c) with the addition of fullerene (C_60_) nanoparticles; and (d) a trivalent chromium (Cr^3+^) electrolyte with C_60_ addition. All coatings showed an increase in microhardness compared with the metal substrate. Trivalent coatings were thinner (10 µm) than the hexavalent coatings (23–40 µm) and resulted in increased roughness and crack density. Wear was found to be reduced for the hexavalent chromium coatings by 70–84% compared with the CoCr–CoCr reference bearing while the trivalent chromium coating even increased wear by more than 300%. The addition of fullerenes to the electrolyte did not show any further tribological effect.

## 1. Introduction

In a historic approach, hard-on-hard bearings for total hip arthroplasty have gained increasing interest after identification of the ‘particle disease’ provoked by the biological potential of polyethylene wear in the late 1980s [1,2]. The term ‘hard-on-hard’ is based on the hardness of the articulation materials, which are significantly increased compared with the conventional or cross-linked polyethylene. As a consequence, ceramic-on-ceramic (CoC) as well as all-metal CoCr bearings (metal-on-metal; MoM) have been widely used for young patients in particular where early aseptic loosening is expected after replacement with polyethylene bearings. In 2007, the National Joint Registry for England, Wales, Northern Ireland, and the Isle of Man (NJR) reported that over 20% of all implanted bearings were MoM bearings [3]. Besides metal-on-polyethylene (MoP, about 60%), MoM was therefore the second-most-used bearing type at that time in the United Kingdom due to excellent in vitro results from simulator studies [4,5]. This number decreased dramatically after high failure rates were reported for single resurfacing implants and reports of an increased incidence of revision with larger stemmed metal-metal combinations (≥32 mm) [6,7]. Following these findings, several health authorities published safety recommendations on the use of all-metal bearings and advised a careful introduction of new devices, as well as a strongly restricted use of large head MoM articulations [8,9,10,11]. As a result, all-metal bearings have widely and for the most part been abandoned in the orthopedic community [6,12], even though reasonably good outcomes are reported in single clinical centers who continue to use small head MoM systems or hip resurfacing [13,14,15]. It has therefore been concluded that these material combinations may work well under well-defined conditions, but are prone to malpositioning of the components [16], and are limited in implant designs [17,18] and tolerances [19]. In particular, hip resurfacing should not be used by low-volume surgeons [20] and only under consideration of a well-chosen patient selection and surgical technique [21,22]. Increased wear of MoM bearings is associated with adverse local tissue reactions (ALTR) [23,24] and in this context, metal ion levels, in particular those of cobalt, are used as an indicator for the need of close follow-up or urgent intervention [9,25].

Based on the in vitro as well as clinical long-term experiences, MoM bearings still have the potential to be used as a bearing option in total hip arthroplasty, but need to be considered carefully. As critical factors, implant design, positioning, and also the low-wearing properties of the material are currently the main focus points. From this perspective, metal coatings represent a technically interesting approach, which can alter the tribological properties.

The electrochemical co-deposition technique represents an established method for the development of new metal-based composite materials. The properties of the coating can be optimized for the specific application by the addition of hard or lubrication particles. However, this is not easily realized for each base coating, e.g., nickel is known as a very good base material for a variety of co-deposition coatings, which can be economically produced on a larger scale. In the field of hexavalent chromium-based co-deposition coatings, hard alumina oxide or diamond particles in the size range of 2–5 µm or the size of 0.5 µm, respectively, are currently embedded in a chromium matrix by an iterative polarity reversal process [26]. In addition, deposition of decorative chrome coatings on the basis of a trivalent chromium electrolyte has been realized. Trivalent chrome deposition was developed to reduce environmental burden and is intended as a new alternative for co-deposition of particles within this coating because the electrolyte does not oxidize to the same extent as hexavalent chromium and the ion strength is not as high. However, decorative chromium layers are generally not thicker than 5 µm.

Besides graphite and diamond, fullerenes are the third carbon allotrope. These were discovered in 1985 and consist of sixty carbon atoms (C_60_) in a highly symmetric closed structure [27]. Comprehensive research has been conducted on the potential medical application of pristine fullerenes and a variety of derivates, e.g., water-soluble forms used as a contrast agent [28], or fullerol, (C_60_(OH)_x_) which has been investigated as a tumor inhibitor [29]. As a nanomaterial, fullerene toxicity and biocompatibility are considered to be critical factors, and these have been scientifically addressed by numerous in vitro and in vivo studies. In that context, it has been shown that the biological effect strongly depends on the structure of the fullerene nanoparticles [30]. Diverse animal models have been used to study the fullerene absorption through different pathways. Increased fullerene exposure in the atmosphere revealed only transient inflammation in rodents but potential tissue damage at higher doses [31,32,33]. In vitro and in vivo genotoxical examination did not reveal negative effects when a C_60_ nano-suspension was used [34]. Intraperitoneal injection of solubilized fullerenes and derivates to mice and rats caused accumulation in diverse organs without inflammatory reaction [35,36]. Several studies have shown that nano-sized C_60_ are delivered all over the body [37,38]. Even though discussions about nano-sized particles crossing the blood-brain barrier are still ongoing [39], no fullerene molecules have been found in the brain. Based on these results, pristine C_60_, as used in the present study, seems to show no acute toxicity in an animal model [30,40].

The present study is based on the hypothesis that fullerenes may act as a nano-bearing when incorporated into a chromium matrix by increasing the hardness of the articulating surfaces and using their ability to freely rotate in their crystal structure [41]. The aim of this study was, therefore, to use electroplating of chromium coatings with and without co-deposition of fullerene particles in order to decrease wear and the quantity of ions released during articulation.

## 2. Results

### 2.1. Characterization of the Coatings

**Figure 1:**
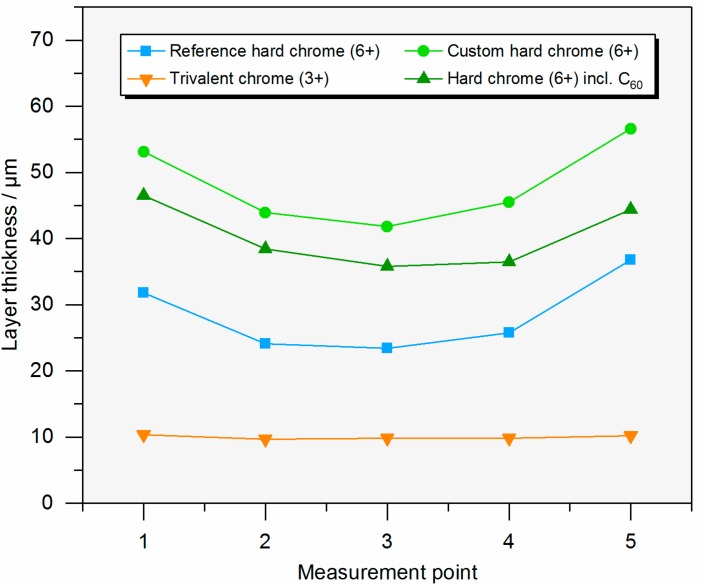
Profile of the layer thickness along the cross section (one sample of each group). Measurement points are equally distributed over the coated width of the sample.

**Figure 2:**
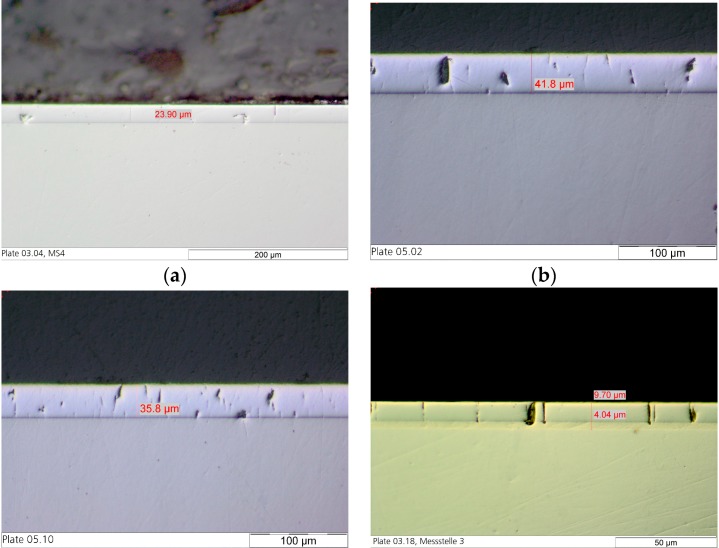
Cross sections of the different testing series for tribological analysis: (**a**) Reference hard chrome (6+); (**b**) Custom hard chrome (6+); (**c**) Hard chrome (6+) incl. C_60_; and (**d**) Trivalent chrome (3+).

**Figure 3:**
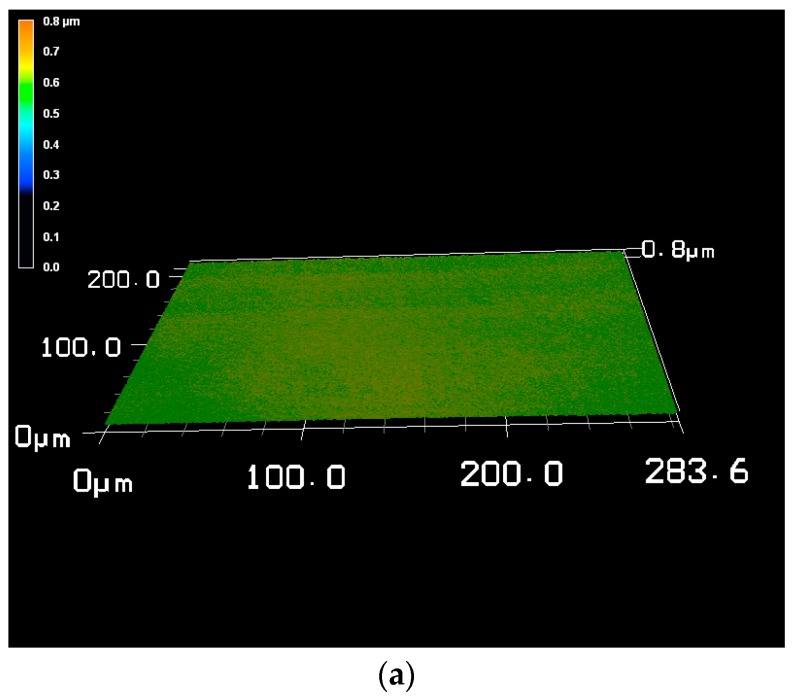

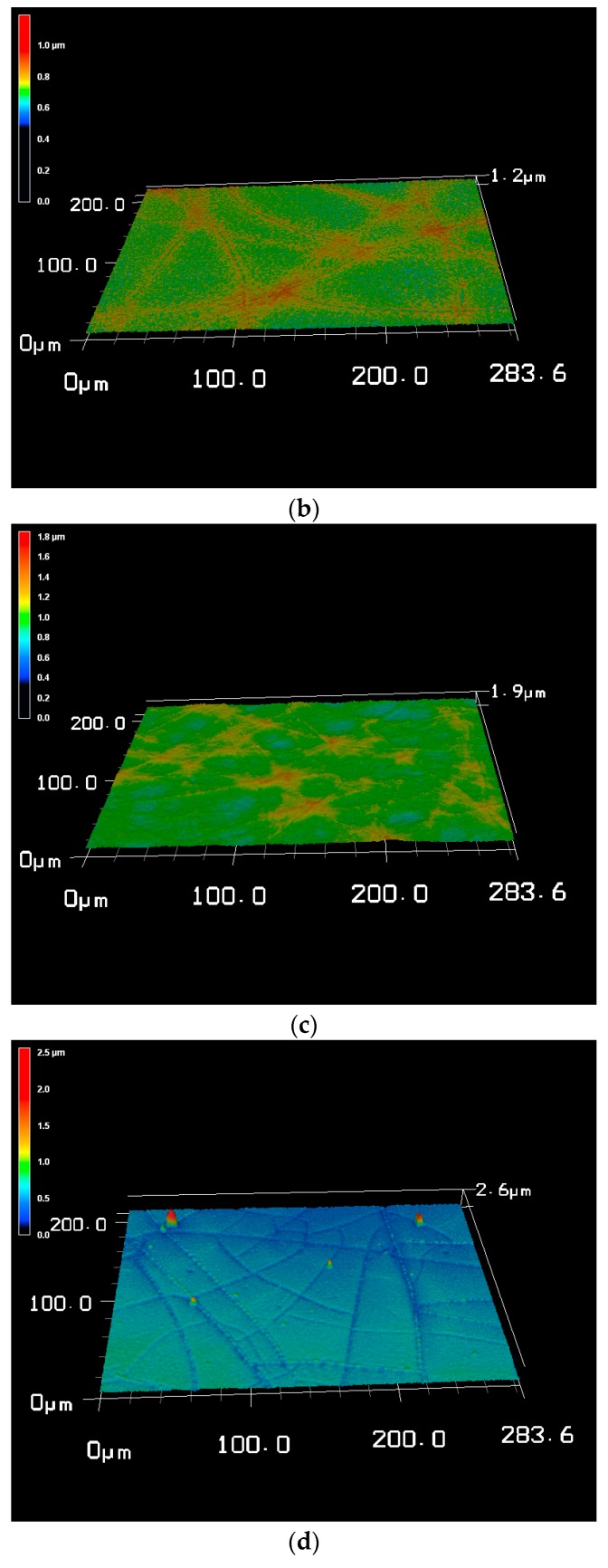
3D profile of the surface: (**a**) polished CoCr substrate; (**b**) Reference hard chrome (6+); (**c**) Hard chrome (6+) incl. C_60_; and (**d**) Trivalent chrome (3+).

**Figure 4:**
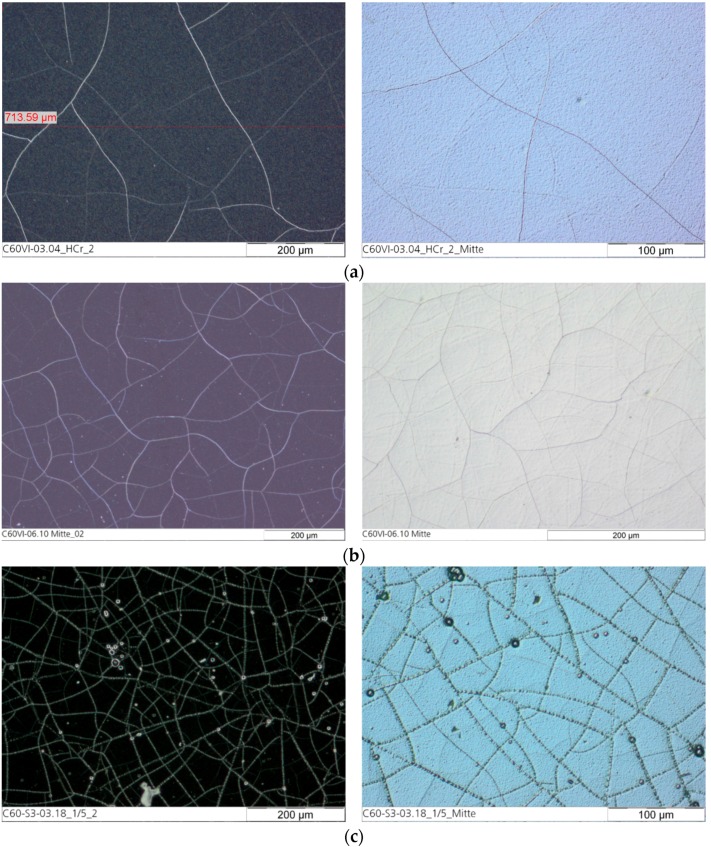
Surface of the chromium layer as dark field picture (left) and DIC picture (differential interference contrast; right): (**a**) Reference hard chrome (6+); (**b**) Hard chrome (6+) incl. C_60_; and (**c**) Trivalent chrome (3+).

Table 1 shows the results of the characterization of the different coatings. The layer thickness and the current efficiency are dependent on the plating parameters. The current efficiency of the custom hard chrome (6+) electrolyte with a CrO_3_ content of 50 g/L was slightly higher than that of the industrial reference electrolyte (CrO_3_: 280 g/L) while the current density was decreased. In standard hard chrome deposition, the current efficiency increases with the current density. Figure 1 and Figure 2 show the thickness profile along the samples showing the typical distribution of the layer thickness due to the electric flux line. The microhardness of the hexavalent coatings was about 900–950 HV0.025 (Vickers hardness) where no difference is seen between the electrolytes and the presence of fullerene particles. The hardness measurement of the trivalent chrome coating was difficult to carry out because the layer was very thin and brittle. The number of cracks in the surface of the coating from the custom electrolyte with a reduced CrO_3_ content of 50 g/L was slightly higher than from the industrial reference electrolyte. Most cracks were found on the coating from the trivalent chrome (3+) electrolyte, where the surface roughness was also increased compared with the coatings from a hexavalent electrolyte (Figure 3). However, no relevant difference in crack density between the coatings from the hexavalent electrolytes was reported. It must be noted that the coating from a hexavalent electrolyte with a CrO_3_ content of 50 g/L was thicker than that from an analogous electrolyte with a CrO_3_ content of 280 g/L, and the roughness was increased with the thickness. The surface roughness of the trivalent chrome layer was higher than those of all hexavalent chromium coatings. One reason for this could be that the decreased process stability of the trivalent process and the smaller thickness of the trivalent process were optimized for decorative plating with a layer thickness of less than 1 µm. The number of cracks in the coatings, measured on the surface, strongly depends on the plating parameters (Figure 4). The reference hard chrome (6+) coating showed the fewest cracks, while the coating from the trivalent chrome (3+) electrolyte had the highest crack density (about 300 cracks/cm). There seems to have been no effect from the addition of fullerenes on the number of cracks at the surface. 

### 2.2. Detection of the Fullerenes

**Figure 5:**
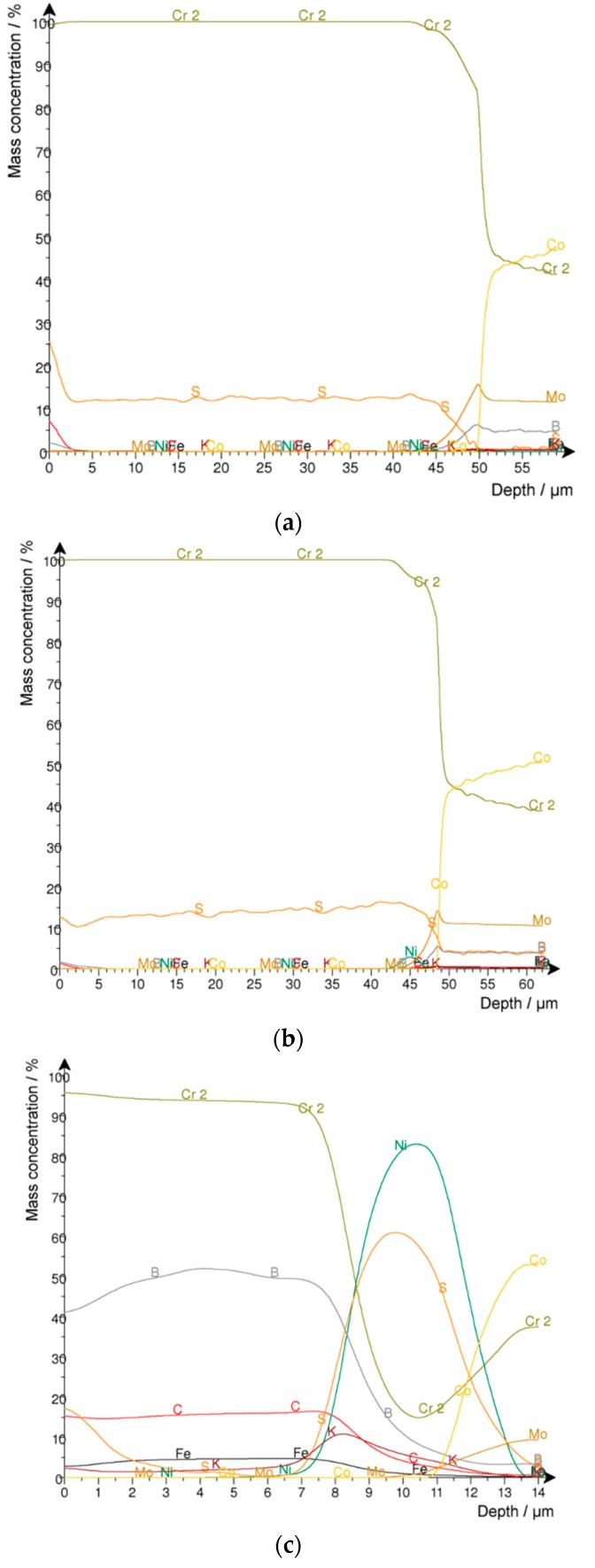

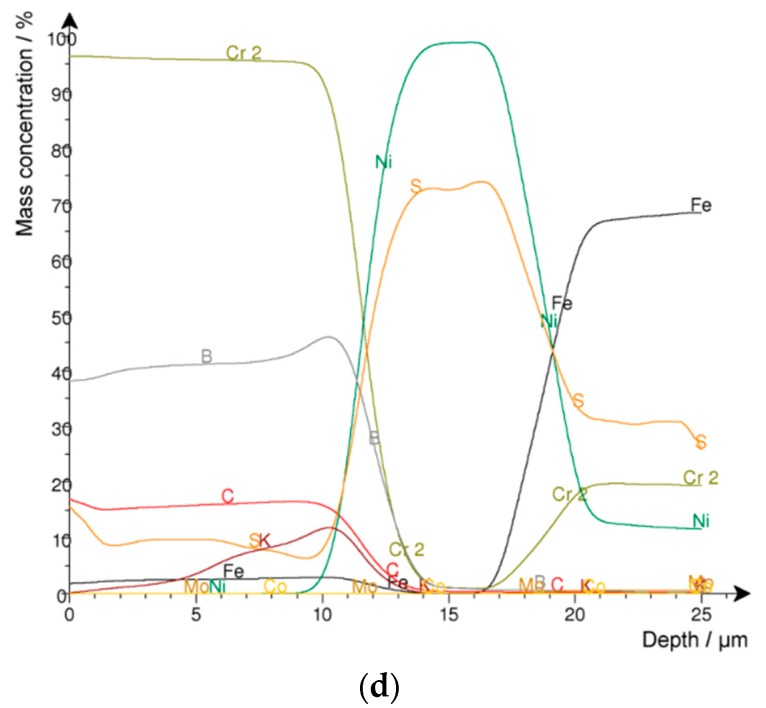
Glow Discharge Optical Emission Spectroscopy (GDOES) depth profile (Fe-s(100%), C-s(10%), Cr-s(100%), Ni-s(100%), S-s(0.1%), B-s(0.1%), K-s(0.1%). Mo-s(100%), Co-s(100%)): (**a**) Custom hard chrome (6+); (**b**) Hard chrome (6+) incl. C_60_; Trivalent chrome (3+) (**c**) with and (**d**) without fullerene (C_60_) addition.

A quantitative Glow Discharge Optical Emission Spectroscopy (GDOES) depth profile analysis was executed (Figure 5). In this case, the carbon content in the different chromium layers was of particular interest. Hard chrome coatings from the hexavalent electrolyte (CrO_3_: 50 g/L and HTAB) with and without fullerenes were analyzed as a reference. In both series, no carbon was detected. On the other hand, carbon was found in the coatings from the trivalent chrome electrolyte. However, there was almost no difference between the carbon content with (1.35–1.55 wt %) and without (1.55–1.6 wt %) fullerene addition to the electrolyte, which may be explained by the use of the graphite anode during electroplating from a trivalent chrome (3+) electrolyte. Therefore, it was not possible to detect the co-deposition of fullerenes in the chromium layer.

### 2.3. Wear Testing

**Figure 6:**
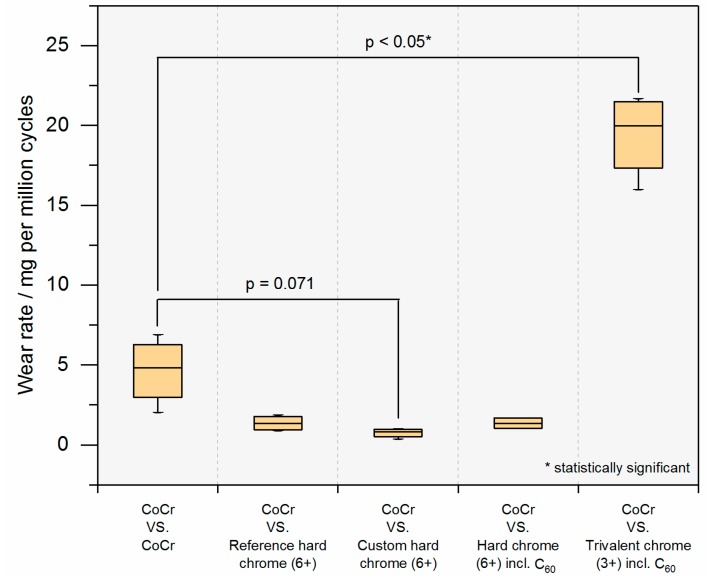
Plate wear of non-identical bearing material combinations compared with the reference CoCr metal-on-metal (MoM) bearing (note: pin wear given separately, see Table 2).

Figure 6 shows the wear results of the coated plates which articulated against a non-coated CoCr pin. Coating of the plates from a hexavalent chromium electrolyte resulted in a clear but non-significant reduction in wear compared with the reference bearing (CoCr–CoCr), while coating from the trivalent chrome (3+) electrolyte including C_60_ showed a significant increase (*p* < 0.05) in gravimetric wear. When comparing the different hexavalent coatings, no significant difference was reported (*p* = 0.086), even though the custom hard chrome (6+) coating without fullerene co-deposition (based on a hexavalent electrolyte with a CrO_3_ content of 50 g/L) performed the best. Pin wear is given in Table 2.

**Figure 7:**
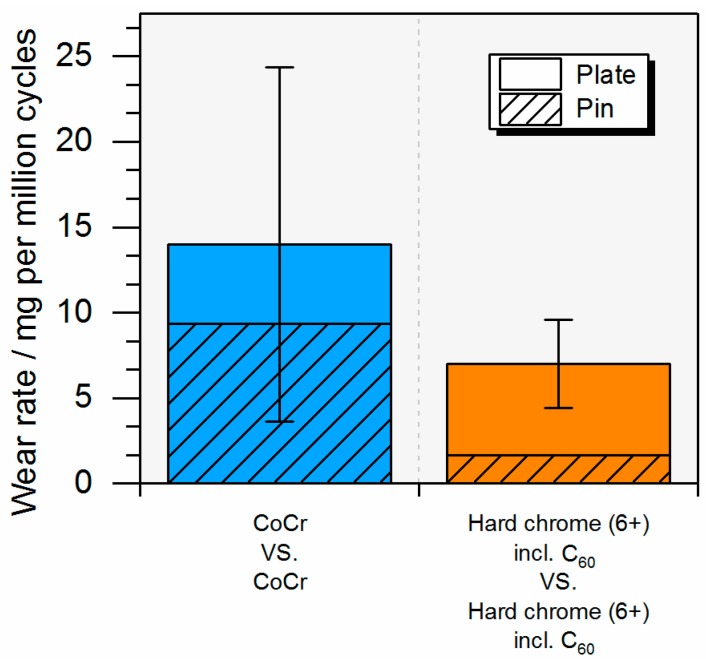
Combined pin and plate wear of bearings with same articulating partners.

For the bilaterally coated bearings, in which both articulating components—pin and plate—were coated, a lower but not significant (*p* = 0.24) total wear rate was seen (7.0 ± 2.6 mg/10^6^ cycles) compared with the conventional all-CoCr MoM reference bearing (14.0 ± 10.4 mg/10^6^ cycles) (Figure 7).

**Figure 8:**
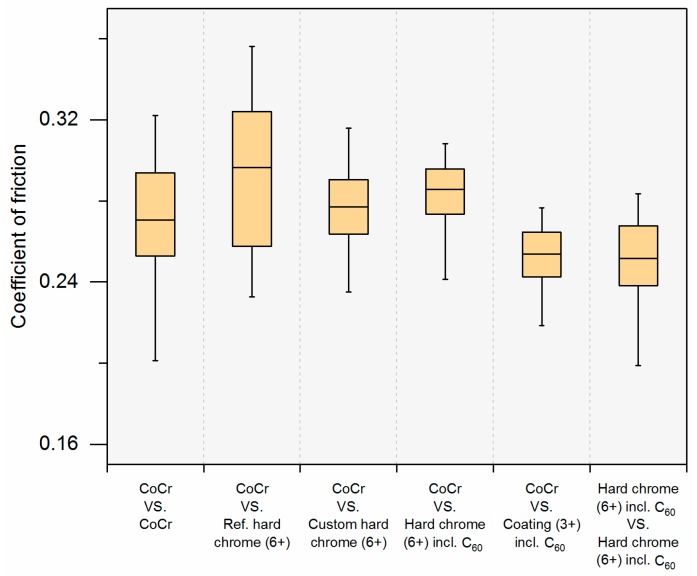
Mean friction coefficient over time (*n* = 1); differences between the investigated bearings.

As expected, a dynamic evolution of the resultant frictional force over one cycle was observed. However, the mean coefficient of friction differed only slightly between the bearings but seemed to be relatively high in all cases (Figure 8). No effect of fullerene addition is seen.

**Figure 9:**
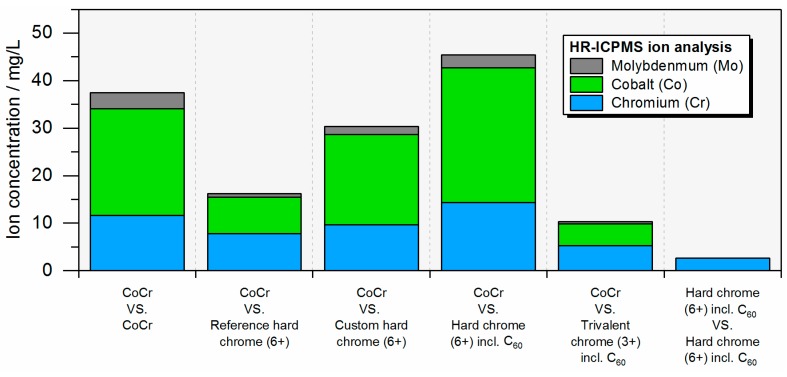
Cumulative ion release after end of test (one million cycles) of chromium (Cr), cobalt (Co), and molybdenum (Mo) ions (HR-ICPMS: High Resolution Inductively Coupled Plasma Mass Spectrometry).

In clinical practice, ion release is currently of interest for metal-based bearings and so the cumulative ion concentration after the total of one million cycles was calculated. It is interesting to notice that chromium ion concentration was lowest for the bilaterally coated bearings where no cobalt or molybdenum ions were detected (Figure 9), showing that the CoCr substrate material did not wear off during articulation. For the unilaterally coated bearings, for which a non-coated CoCr pin articulated against a coated plate, no correlation with the plate wear volume (Figure 6) was observed. This is due to the effect of pin wear, which is given in Table 2.

## 3. Discussion

The tribological testing showed an increase in the wear resistance of the hexavalent chrome (6+) coatings and a reduction to about one third of the wear measured for the CoCr MoM reference bearing. These findings are supported by earlier studies investigating the tribological behavior of chromium-based coatings, e.g., CrN (reduced wear of CrN–CrN by 80–86% compared with CoCr MoM) [42,43,44,45]. This may be due to the higher hardness of the coating compared with the CoCr substrate, increasing the wear resistance, which is furthermore supported by the low wear rates and increasing ion concentration (released by the CoCr pin) of the hexavalent chromium coatings. In this context, the co-deposited hard chrome–fullerene coatings also showed higher hardness and an increase in ion release due to wear of the CoCr pin. Bilaterally coated bearings therefore showed a very low wear rate and cumulative chromium ion release while cobalt and molybdenum traces from the substrate were not measured. In contrast, co-deposition based on a trivalent chrome (3+) electrolyte resulted in a considerable increase in gravimetric wear. On the other hand, total ion release was reduced compared with the reference due to a considerably lower wear of the CoCr pin against the trivalent chrome (3+) plate that is apparent from Table 2. There was no correlation between plate surface roughness and wear rate or ion release.

The positive effect of fullerenes co-deposited with hard chrome coatings hypothesized for this study cannot be confirmed, as the wear-reducing effect is not clearly linked to hypothetic fullerene incorporation. In both cases, with and without fullerene additives against a CoCr pin, comparable wear rates and friction coefficients were measured. It was shown that the presence of nano-scaled particles embedded in electrochemical coatings was not evident, in particular in the case of carbon. However, it is possible to modify the coating structure and properties that are directly linked to the presence of the hard particles in the electrolyte. This has also been reported by other studies, e.g., an increase in coating hardness with the addition of silicon dioxide nano-powders [46]. In that context, the biological potential of nano-scaled materials, e.g., carbon nanotubes [47], is still under discussion, and it seems that not only the carbon material, but also its structure are relevant as an acute adverse reaction is described to be more likely for carbon nanotubes than fullerenes [40,48]. However, since the toxicity of pristine C_60_, as used in this study, has not yet been deduced, it should be handled under the precautionary principle.

MoM bearings have nearly disappeared from the market [6,49], although metals present advantageous properties that make them suitable to be used as a bearing material in total joint arthroplasty, such as high fracture toughness, low wear under well-defined conditions, and long clinical experience [5,50]. Regarding the ion release due to a tribological and corrosive attack, coatings present a technically challenging approach that involves altering the tribological contact while keeping the beneficial properties of the metal substrate. In this context, titanium nitride (TiN) and oxidized zirconium (both about 5 µm in thickness) are the best known surface modifications used in the orthopedic field [51]. Due to the protection of the underlying CoCr substrate, these surface modifications are particularly used for patients who suffer from metal allergy or hypersensitivity [52]. Even though TiN was introduced in the late 1980s, scientific literature in this field is still rare. However, there are several reports on failure of these hard surface layers, which get sharp-edged and can, in turn, increase the wear of the polyethylene counterpart [53,54,55]. Two properties must therefore be ensured by a potential coating for orthopedic bearings: (1) low wear (in a hard-on-hard configuration or against polyethylene), and (2) adhesion on the metal substrate needs to be guaranteed. The present study focused on the tribological performance of fullerene co-deposition and has shown the potential for reducing wear in a hard-on-hard configuration. No coating failure due to wear testing was observed. Future steps will be needed to identify the wear rate against the softer polyethylene, either in its conventional or cross-linked form, and to evaluate coating adhesion.

The present study has identified the potential of chromium coatings with and without fullerene co-deposition, which were applied using an electrochemical approach. In a pin-on-plate screening test setup, coatings from a hexavalent chrome (6+) electrolyte clearly reduced wear of the plates and showed less total ion release compared with conventional CoCr MoM bearings when used without the fullerene additives. Hard-on-hard bearings with both articulating partners coated with a fullerene co-deposited chromium layer showed promising tribological wear rates while drastically reducing ion release. Furthermore, besides biotribological applications in artificial joints, these bearings may also be interesting in the larger field of general mechanical engineering.

## 4. Materials and Methods

### 4.1. Electrolytes and Parameters

The final composition of the electrolytes and the plating parameters are based on screening tests with different concentrations and wetting agents. In the hexavalent chromium electrolyte, the following surfactants were investigated: the cationic surfactants Acetylcholin Chlorid (ACh) and Hexadecyl Trimethyl Ammonium Bromide (HTAB), and the nonionic surfactants Polyethylene Glycol 400 and Tween 20 (all VWR International GmbH, Germany). In this study, HTAB was used since it was stable in the electrolyte over time and the fullerenes did not agglomerate. Based on the deposition from a trivalent chrome (3+) electrolyte, the anode material was varied between graphite, platinum-plated titanium, mixed-oxide-plated titanium, and a diaphragm anode to evaluate the best deposition quality. The cathode materials for screening tests were brass sheets and stainless steel plates (1.4301, X5CrNi18-10).

The chromium and chromium-fullerene coatings were electrochemically deposited from hexavalent and trivalent chromium electrolytes. According to the test protocol, five surface conditions were taken into account for further analysis:

(a) **CoCr:** uncoated and polished CoCr which serves as a clinical reference;
(b) **Reference hard chrome (6+):** hard chrome coating based on an industrially available standard electrolyte with a CrO_3_ content of 280 g/L;
(c) **Custom hard chrome (6+):** hard chrome coating based on a reduced CrO_3_ content of 50 g/L;
(d) **Hard chrome (6+) incl. C_60_:** same as (c) but including 0.75 g/L of HTAB and 0.375 g/L of fullerenes (C_60_);
(e) **Trivalent chrome (3+) incl. C_60_:** industrially available trivalent chromium electrolyte (pristine trivalent chrome (3+) without C_60_ used for coating characterization).

The constituents of the custom chrome (6+) electrolyte for the co-deposition of fullerene were CrO_3_ (50 g/L), H_2_SO_4_ (0.5 g/L), HTAB (0.75 g/L), and C_60_ fullerenes (0.375 g/L; BuckyUSA, Houston, TX, USA). The trivalent chrome (3+) electrolyte was an industrial decorative chromium electrolyte (Trichrome^®^ Plus, Atotech Deutschland GmbH, Berlin, Germany). The hard chromium layers on the reference plates were deposited from an industrial standard chromium electrolyte consisting of CrO_3_ (280 g/L) and H_2_SO_4_ (3.36 g/L) (CrO_3_:H_2_SO_4_ = 100:1.2). The electrolytes were continuously monitored for hexavalent and trivalent chromium and sulfate content by titration and ion chromatography. It was not possible to control the content of the fullerenes. The electrodeposition was carried out galvanostatically using a DC power supply (SM 18–50, Delta Elektronika, Zierikzee, The Netherlands). The electroplating parameters are given in Table 3 and are identical for the coatings with and without the addition of fullerene nanoparticles.

Without addition of a surfactant, the fullerenes tended to agglomerate in the hexavalent chromium electrolyte. Thus, agglomeration was minimized by the addition of HTAB dissolved in 50 mL of deionized water at a constant temperature of 55 °C and stirring by a magnetic stirrer at 600 rpm for one hour. Afterwards, the solution was added to the electrolyte and the electroplating process was started.

The anode was lead for the hexavalent chrome (6+) deposition and graphite for the trivalent chrome (3+) deposition. The substrate (cathode) and the pin for tribological testing were made of a polished CoCr steel (according to ISO 5832-12) with an initial surface roughness R_a_ of 0.03 µm. The effective plating area was 0.143 dm^2^.

Before electrodeposition, the substrates were cleaned with acetone, hot degreased (5 min), electrolytic degreased (30 s), and deoxidized in 5 % H_2_SO_4_ (30 s). The samples were rinsed between each cleaning step and at the end of the process. After cleaning, a thin nickel-strike layer was deposited to enhance the adhesion on the stainless steel. For the tests where both pins and plates were coated, a second nickel layer (bright nickel) was applied between the nickel-strike and chrome with a thickness of 15–20 µm.

### 4.2. Experimental Setup for Electrodeposition

**Figure 10:**
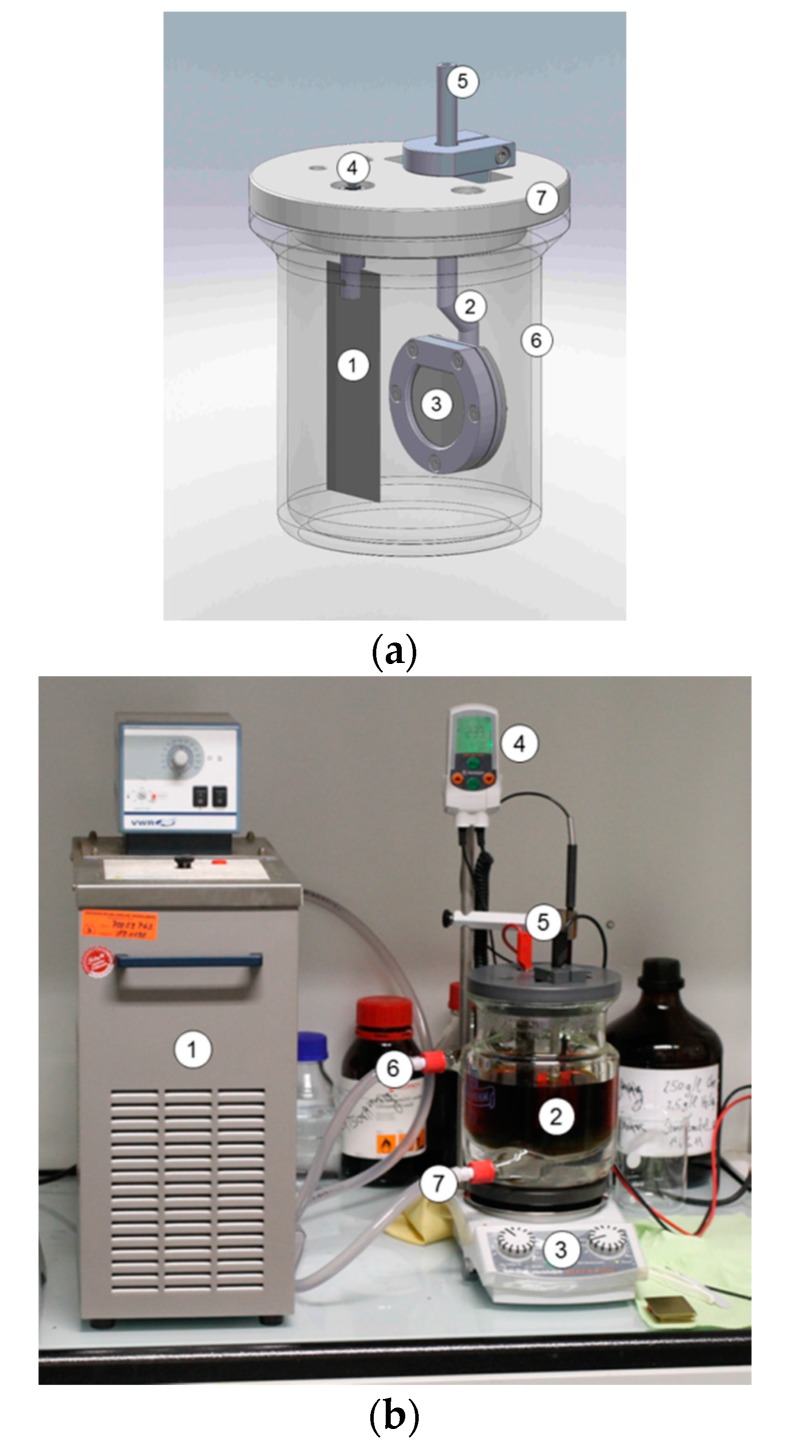
Setup for electroplating: (**a**) detailed component view with (1) anode, (2) cathode holder, (3) cathode, (4) anodic contact, (5) cathodic contact, (6) double-walled glass beaker, and (7) cover plate; (**b**) experimental setup with (1) heating-cooling unit, (2) glass beaker with electrolyte, (3) magnetic stirrer, (4) thermometer, (5) contacts, (6) cooling water outlet, and (7) cooling water inlet.

The electrodeposition was realized in a custom-made plating setup with an electrolyte volume of 750 mL. As the plates did not have any threads for contacting, a special holder was designed (Figure 10). The sample was clamped in the holder and contacted on the back side. A double-walled glass beaker was used for stabilization of the electrolyte temperature (heat removal), as temperature increases during plating due to the applied energy in the electrolyte. The plating bath was heated and stirred on a magnetic stirrer.

### 4.3. Characterization of the Coatings

The coatings were metallographically characterized. Cracks on the surface were counted with a light microscope in the dark field (Axioplan microscope, Zeiss, Jena, Germany; microscope camera, Olympus, Hamburg, Germany). The layer thickness and the microhardness were measured in the cross section after cutting the plates (Secutom 10, Struers, Willich, Germany). The microhardness was obtained using a Vickers indenter with a load of 25 p (pond) or 10 p for 10 s (MHT-4 mircohardness tester attached to an Olympus microscope). The surface roughness measurements were taken with a Color 3D Laser Scanning Microscope (VK-8700, Keyence, Osaka, Japan). The measurements were taken at three different positions in the center of the sample with 50× magnification and a z-step size of 0.2 µm. The current efficiency η is calculated by:

(1) $$\eta =\frac{\rho \times d\times n_{e}\times F}{j\times t\times M}$$

where ρ is the density in g/cm^3^ (=7 g/cm^3^), *F* is the Faraday constant in As/mol (=96,487 As/mol), *n_e_* is the number of electrons, *d* is the layer thickness in dm, *j* is the current density in A/dm^2^, and *M* is the molar mass in g/mol (=51.9961 g/mol).

Co-deposition of the fullerenes was controlled by GDOES (Glow Discharge Optical Emission Spectroscopy). From each sample, two GDOES depth profile analyses were performed where Ar5.0 was used as the sputtering gas. The measurements were carried out with a constant voltage of 1100 V and a regulated current of 10 mA. Under these conditions, the detection limit was 0.3 and 32 ppm.

### 4.4. Wear and Friction Analysis

**Figure 11:**
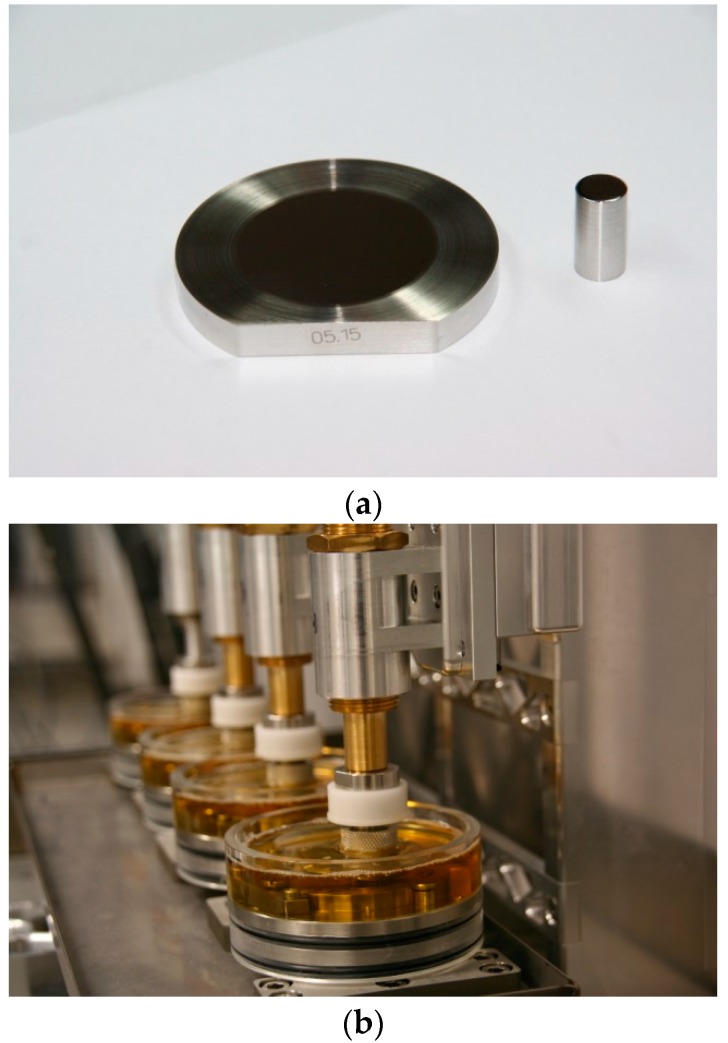
Tribological testing setup: (**a**) pin and plates used for wear and friction measurements; (**b**) pin-on-plate tribometer with four wear stations where station #1 is equipped with an additional friction measurement unit.

The tribological analysis was performed in a custom-made four-station pin-on-plate simulator (Figure 11) for a total of one million cycles at a frequency of 1 Hz. The specimens’ geometries were adapted to the Hertzian pressure comparable to that in an artificial hip joint (MoM). Therefore, the pins had a radius of 500 mm at the articulating end, resulting in a surface pressure of 46.8 MPa between the pin and the plate at a constant axial force (*F_z_*) of 10 N. Pin and plate articulated on an elliptic path with the length of the major axes of 24 mm and 12 mm as described for the main contact point in the artificial hip bearing [56]. Tests were run at room temperature in diluted bovine serum (PAA Laboratories GmbH, Pasching, Austria) with a protein content of 30 g/L, which is the standard substitute for the synovial fluid during wear testing of artificial hip joints according to ISO 14242-1. Sodium azide (1.85 g/L) and ethylenediamine tetraacetic acid (7.44 g/L) were added to hinder bacterial growth and minimize layers of calcium phosphate. 

During testing, the lateral forces *F_x_* and *F_y_* were continuously measured on station #1. Based on the resulting frictional force $F_{xy}=\sqrt{F_{x}^{2}+F_{y}^{2}}$, the friction coefficient µ is calculated as

(2) $$µ=\frac{F_{xy}}{F_{z}}.$$

Wear of both pins and plates was assessed gravimetrically every 125,000 cycles (Genius ME235S, Sartorius, Germany; repeatability accuracy: ±15 μg). Additionally, metal ion analysis was performed using high-resolution inductively coupled plasma mass spectrometry (HR-ICPMS) at an interval of 250,000 cycles (Element2, Thermo Fisher Scientific, Bremen, Germany; detection limits: 0.03 lg/L for Co, 0.05 lg/L for Cr, and 0.01 lg/L for Mo). Details have been published elsewhere [57].

Based on the outcome of the electrochemical co-deposition screening, six groups were identified for tribological testing (each *n* = 4 specimens) (Table 4). In that context, uncoated CoCr against CoCr (#1), as well as the reference hard chrome (6+) electroplating from the industrially available hexavalent electrolyte (CrO_3_ content: 280 g/L) against an uncoated CoCr pin (#2), served as references. Furthermore, uncoated CoCr pins were tested against coatings from a hard chrome (6+) electrolyte with a CrO_3_ content of 50 g/L without (#3) and with the addition of fullerenes (#4), as well as against coatings from the trivalent hard chrome (3+) electrolyte with fullerenes (#5). In addition, one group where both articulating partners—pin and plate—were coated with the hexavalent hard chrome (6+) electrolyte (CrO_3_: 50 g/L) was investigated (#6). Therefore, comparisons were also made between groups in which a CoCr pin articulated against a coated plate (#2–5) and groups in which both articulating partners were identical (#1 and #6).

### 4.5. Statistics

An ANOVA with repeated measures was used to compare differences in wear rates between groups (post hoc test using LSD correction). For the comparison of the bilaterally coated specimens, a Student’s *t*-test was used. In all cases, a *p*-value of <0.05 was considered as significant. All data is presented with mean ± standard deviation.

## 5. Conclusions

Electroplating of standard CoCr substrates based on hexavalent and trivalent chrome electrolytes was performed with and without the addition of fullerene nanoparticles with the aim of increasing wear resistance of a total hip replacement during articulation. All coatings were characterized regarding microhardness, crack density, roughness, and current efficiency during deposition. Even though the addition of fullerenes did not have a direct influence on these coating properties, wear was low for the plates that were coated from the hexavalent chromium electrolyte with addition of fullerene nanoparticles. However, this effect was not significant in comparison to the electroplating based on electrolytes without any fullerene content. The results support the hypothesis that the electrochemical deposition from a hexavalent chromium based electrolyte increases the wear resistance compared with the standard CoCr–CoCr bearing.

## Figures and Tables

**Table 1:** Results of the characterization of the layer.

Parameter	CoCr (Uncoated)	Reference Hard Chrome (6+)	Custom Hard Chrome (6+)	Trivalent Chrome (3+)
Layer thickness in µm ^1^	-	23	35–40	10
Current efficiency η in %	-	10	~13.5	~14
Microhardness in HV (Vickers hardness)	475–540 HV0.025	900–950 HV0.025	900–950 HV0.025	850–900 HV0.005 ^2^
Number of cracks in cracks/cm	-	~70	~100–120	~300
Surface roughness R_a_ in µm	0.03	0.05	0.06	0.1

^1^ measured at the center of the plate, ^2^ layer is brittle and very thin (effect on hardness measurements).

**Table 2:** Pin wear. Comparison of bearing material combinations (in mg per million cycles).

CoCr vs. CoCr	CoCr vs. Reference Hard Chrome (6+)	CoCr vs. Custom Hard Chrome (6+)	CoCr vs. Hard Chrome (6+) Incl. C_60_	CoCr vs. Trivalent Chrome (3+) Incl. C_60_
9.37 ± 9.14	2.31 ± 1.40	6.41 ± 5.18	10.72 ± 9.99	1.92 ± 1.12

**Table 3:** Electroplating parameters.

Parameter	Custom Hard Chrome (6+) ^1^	Reference Hard Chrome (6+) ^2^	Trivalent Chrome (3+)
Temperature in °C	55	55	35
Current density in A/dm ^2^	25	40	10
pH	<1	<1	2.6 (2.3–2.7)
Time in min	180	90	45

^1^ hexavalent chromium with CrO_3_ 50 g/L, ^2^ hexavalent chromium with CrO_3_ 280 g/L.

**Table 4:** Testing matrix of the investigated tribological bearings (each *n* = 4).

#	Pin	Plate	Comment
1	CoCr	CoCr	Uncoated reference
2	CoCr	Reference hard chrome (6+) ^1^	Industrial hard chrome coating (CrO_3_: 280 g/L) vs. uncoated pin
3	CoCr	Custom hard chrome (6+) ^1^	Chromium coating (CrO_3_: 50 g/L) vs. uncoated pin
4	CoCr	Hard chrome (6+) ^1^ incl. C_60_	Chromium coating (CrO_3_: 50 g/L) with fullerenes vs. uncoated pin
5	CoCr	Trivalent chrome (3+) ^2^ incl. C_60_	Trivalent chrome coating with fullerenes vs. uncoated pin
6	Hard chrome (6+) ^1^ incl. C_60_	Hard chrome (6+) ^1^ incl. C_60_	Chromium coating (CrO_3_: 50 g/L) with fullerenes, both pin and plate coated

Substrate has been coated from a ^1^ hexavalent (6+) or ^2^ trivalent (3+) chromium bath.

## Supplementary Files

Supplementary File 1
