# Supplementary figures and images for: Hard Chrome-Coated and Fullerene-Doped Metal Surfaces in Orthopedic Bearings
Source: Materials (Basel). 2017 Dec 20;10(12):1449. doi: 10.3390/ma10121449 (PMC5744384; doi:10.3390/ma10121449)

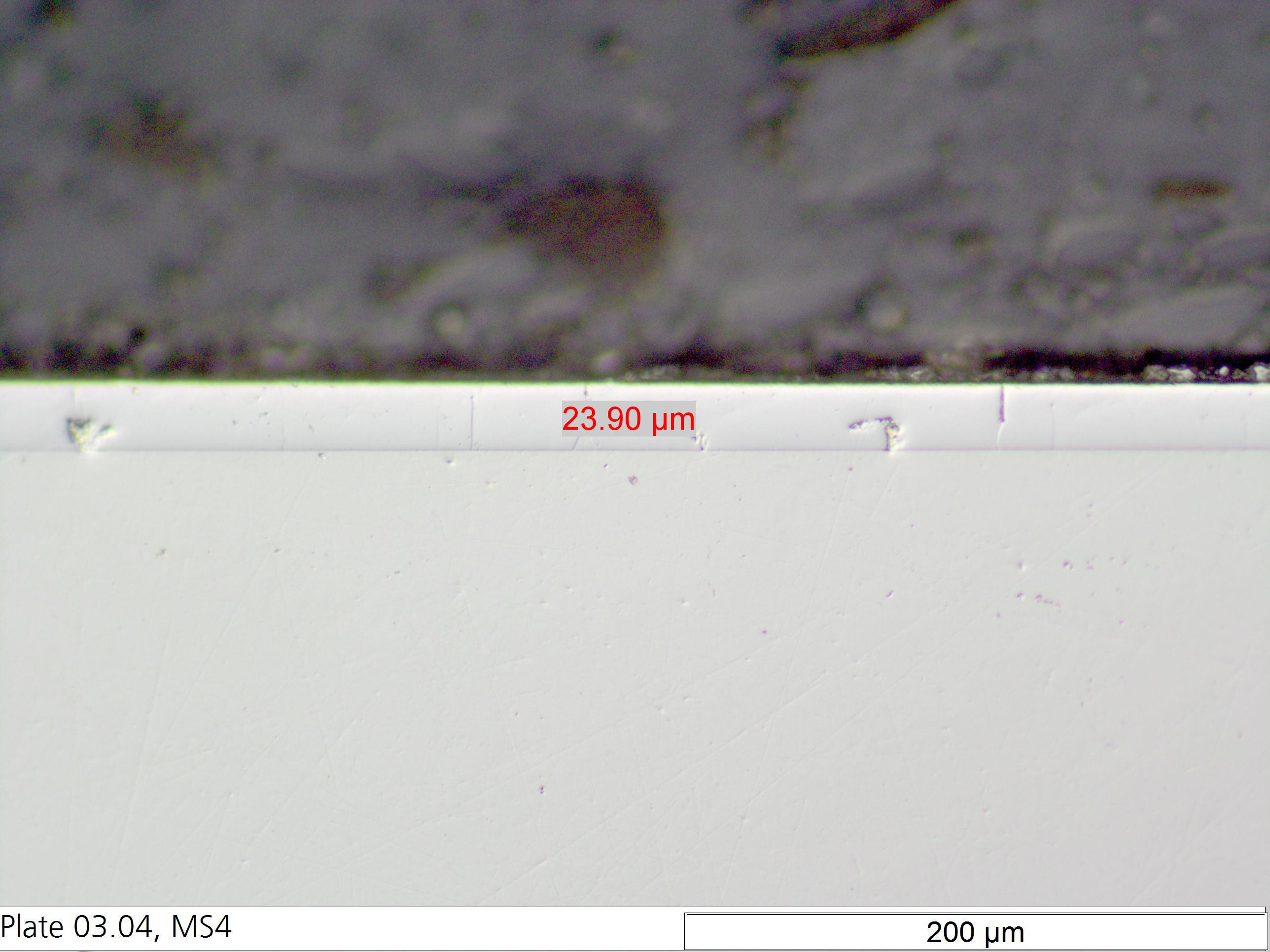

Supplement: Supplementary File 1 [file materials-10-01449-s001.zip › Fig2a.jpg]

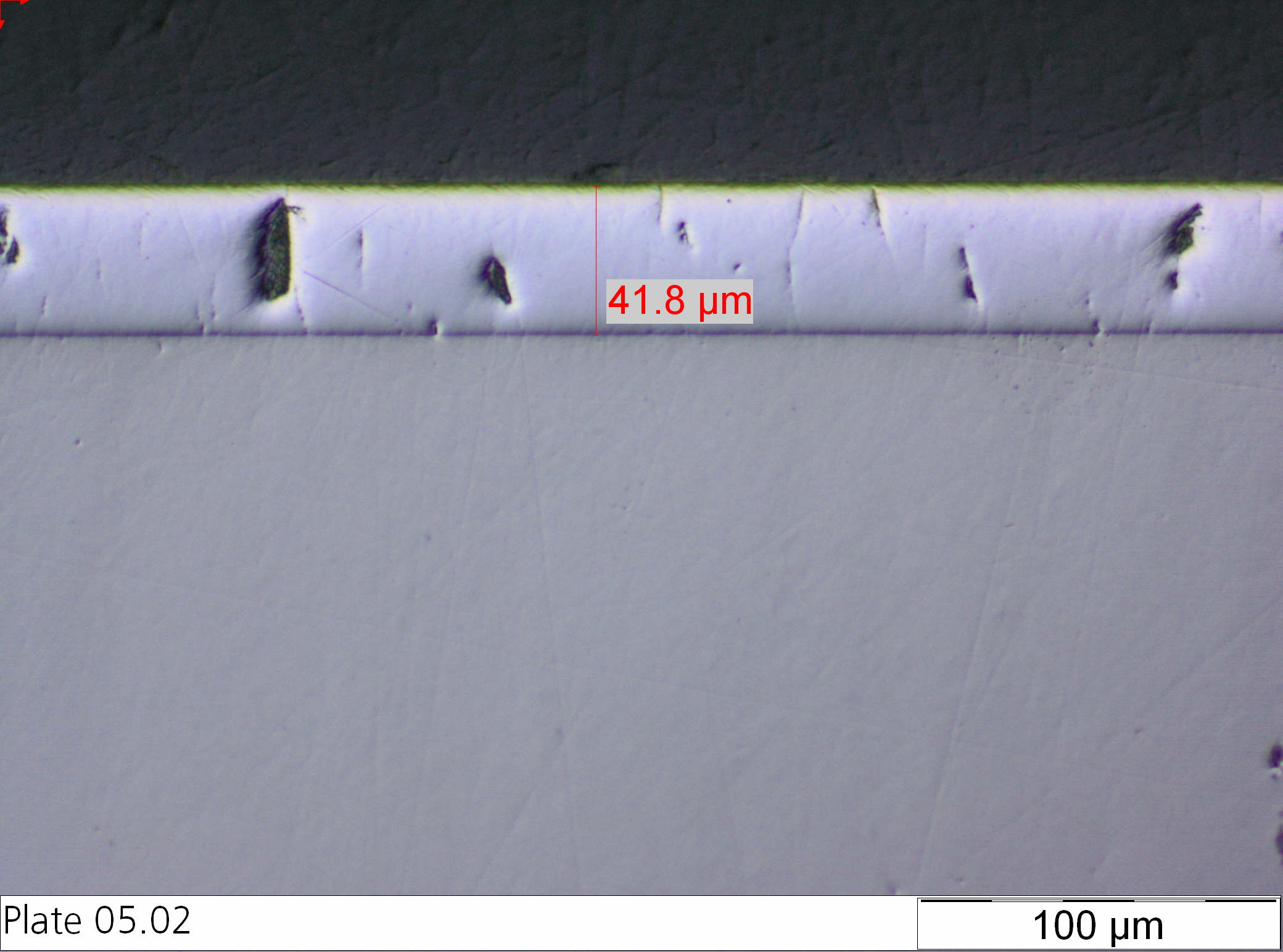

Supplement: Supplementary File 1 [file materials-10-01449-s001.zip › Fig2b.jpg]

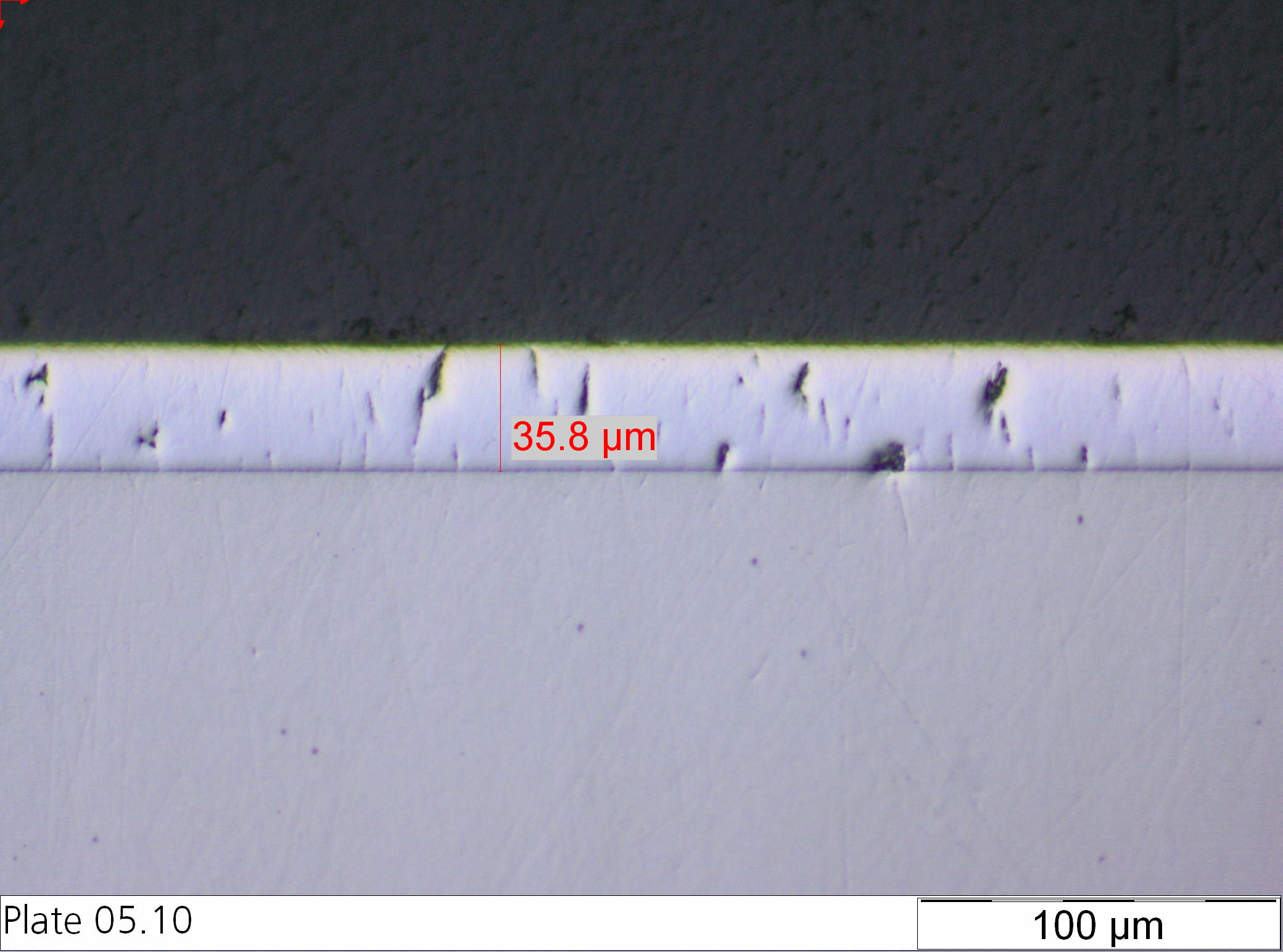

Supplement: Supplementary File 1 [file materials-10-01449-s001.zip › Fig2c.jpg]

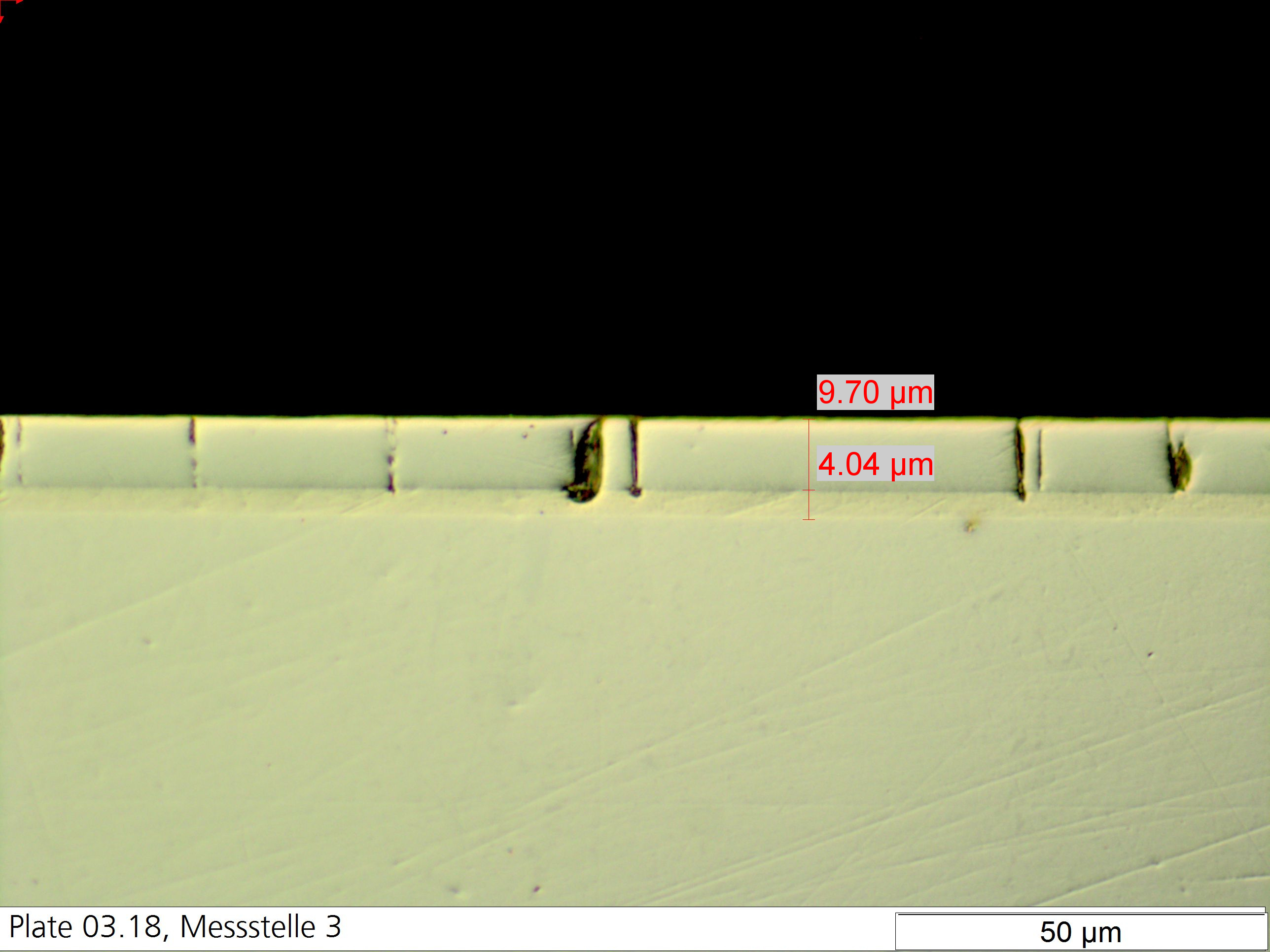

Supplement: Supplementary File 1 [file materials-10-01449-s001.zip › Fig2d.jpg]

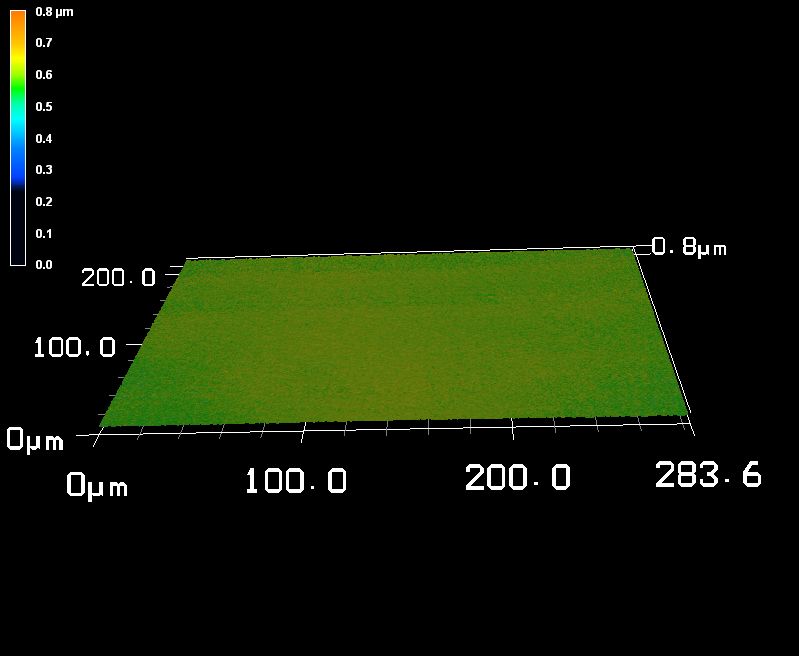

Supplement: Supplementary File 1 [file materials-10-01449-s001.zip › Fig3a.bmp]

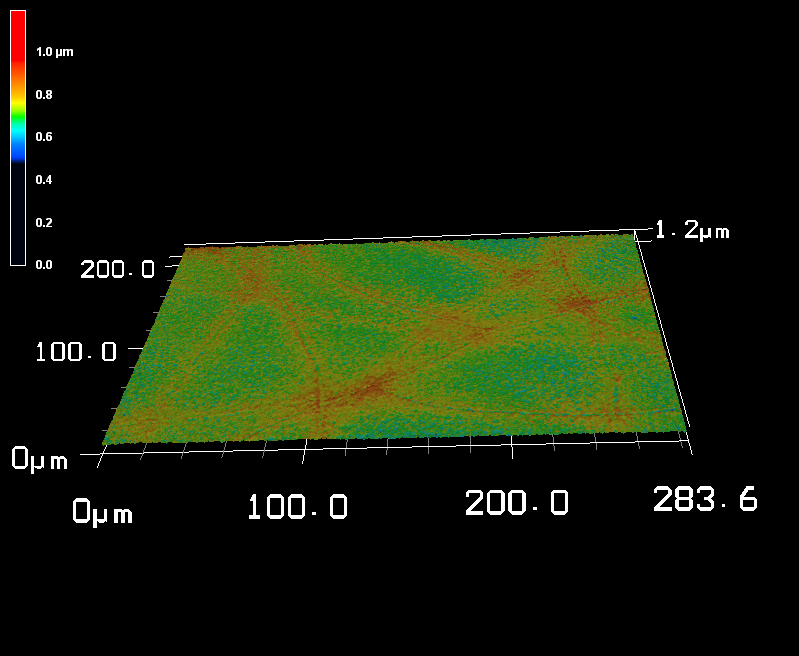

Supplement: Supplementary File 1 [file materials-10-01449-s001.zip › Fig3b.bmp]

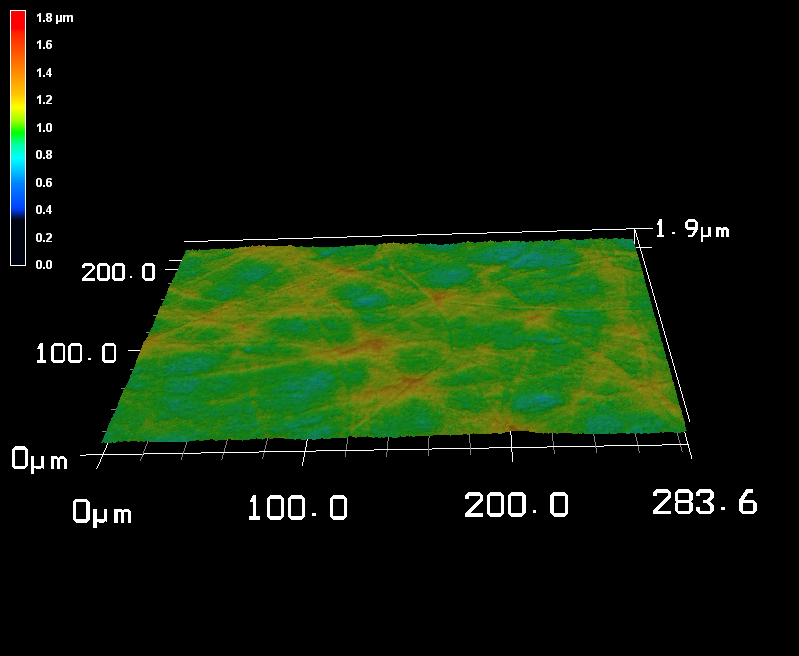

Supplement: Supplementary File 1 [file materials-10-01449-s001.zip › Fig3c.bmp]

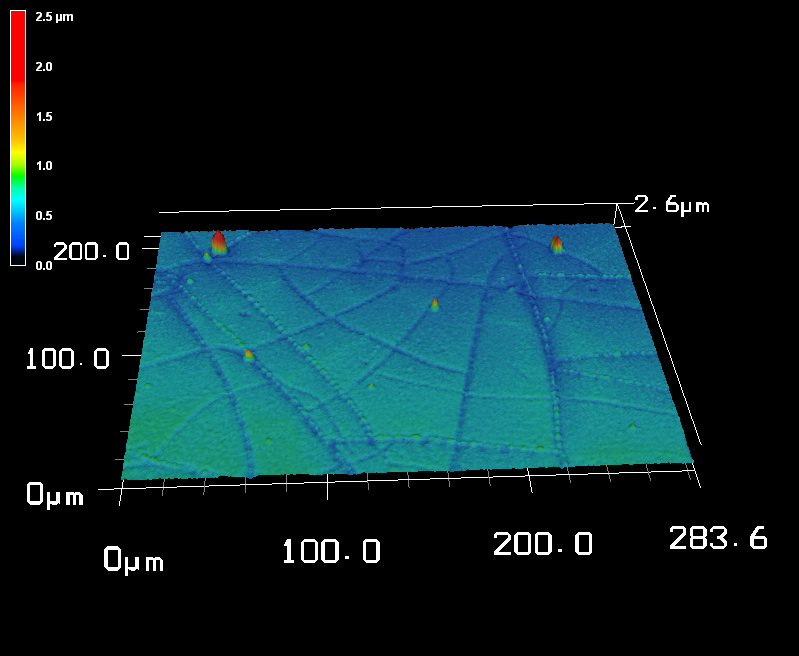

Supplement: Supplementary File 1 [file materials-10-01449-s001.zip › Fig3d.bmp]

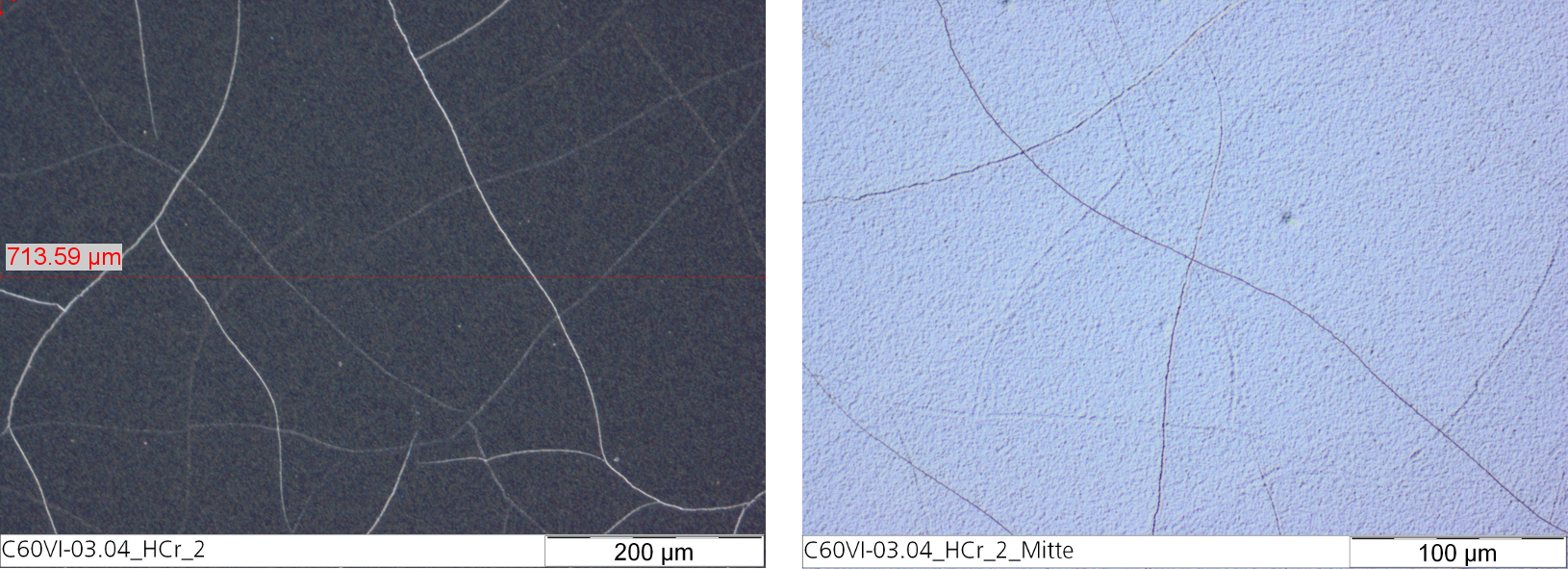

Supplement: Supplementary File 1 [file materials-10-01449-s001.zip › Fig4a.tif]
